# Articulating the need to minimize moral incursions in research: The least infringement condition

**DOI:** 10.1038/s44319-025-00490-w

**Published:** 2025-05-27

**Authors:** Jeremy Sugarman

**Affiliations:** Johns Hopkins Berman Institute of Bioethics, 1809 Ashland Ave, Baltimore, MD 21205 USA

**Keywords:** Economics, Law & Politics, Science Policy & Publishing, Stem Cells & Regenerative Medicine

## Abstract

The “least infringement condition” offers a reasonable articulation of the obligation to use less morally sensitive methods and materials to achieve important scientific objectives.

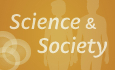

There is an increasing and considerable focus on using alternatives to nonhuman animals in life-science research, including organoid technologies, organ-on-chips, microphysiological systems and in silico models. The interest in developing and using such alternatives—often referred to as “New Approach Methodologies” (Stresser et al, 2024)—is not only evident among scientists and funding agencies, but also regulators (US FDA, 2025). In addition, substantial work on stem cell-based embryo models is advancing scientific understanding of human development while obviating the need to conduct some research using human embryos. Similarly, research with induced pluripotent stem cells often precludes the need to use human embryonic stem cells.

Besides enhancing scientific understanding and sometimes offering opportunities to explore unique scientific questions, these newer technologies and approaches are especially welcome when they can reduce the ethics footprint of research by minimizing what might be called “moral incursions”: using less morally sensitive methods and materials to achieve an important scientific objective. An example of governmental policies aimed at minimizing moral incursions is the strict regulation of research using monkeys and apes in the USA and the EU; another is the legal prohibition of using human embryos in research in some countries. Since such policies are in tension with the right to conduct research and the opportunity to obtain knowledge to be gained from it, it is important for scientists and other stakeholders to have clear definitions and terms concerning when and under which circumstances a moral incursion in research would be justified. This requires a careful analysis of the various principles and conditions that may be employed in the context of decision-making about moral incursions in research.

it is important for scientists and other stakeholders to have clear definitions and terms concerning when and under which circumstances a moral incursion in research would be justified.

Scholars, professional groups, and regulators who deliberate about the ethical acceptability of certain types of research, perhaps not surprisingly, commonly endorse minimizing moral incursions when doing so does not compromise the ability to accurately and effectively answer a particular scientific question. Take for example, research involving organoids and stem cell-based embryo models. Here, research with most types of organoids is generally preferable to using embryo models; non-integrated models are preferable to integrated models; embryo models are preferable to using embryos; and using nonviable embryos is preferable to viable embryos (Perreira-Daoud et al, 2020; Clark et al, 2021; Rivron et al, 2023; ESHRE et al, 2024). This ordering of preferences can be seen to reflect a view about the differential moral status of these entities, privileging the use of those with lower moral status where scientifically feasible so as to minimize moral incursions in research.

In its most recent guidelines, the International Society for Stem Cell Research operationalizes such an approach specifying that there are: “Forms of research with embryos, certain chimeras, and stem cell-based embryo models that are permissible only after review and approval through a specialized scientific and ethics review process. […] All such research should have a compelling scientific rationale and necessitate the use of these materials rather than employ alternative models. The research should use the minimum number of embryos necessary to achieve the scientific objective.” The UK Code of Practice for the Generation and Use of Stem Cell-Based Embryo Model has similar provisions. That is, research that raises moral concerns should only be conducted following independent oversight and approval to ensure that it is scientifically sound and uses morally sensitive materials only to the extent necessary to achieve the scientific objectives. In this way, moral incursions are minimized.

Likewise, there have been longstanding discussions about the need for minimizing moral incursions related to research involving nonhuman animals. The most established framework for doing so was developed by Russell and Burch (1959) in their landmark text, *Principles of Humane Experimental Technique*. The framework describes three Rs: replacement, reduction, and refinement in research with nonhuman animals. While these requirements have been interpreted and defined differently and some view ‘alternatives’ to be synonymous with replacement, the basic set of requirements has had considerable traction among scientists, professional groups, sponsors, regulators, and oversight bodies (Tannenbaum and Bennett, 2015).

A central argument for this approach is the need for humane treatment of animals, which implicitly recognizes the moral status of animals. However, it has been argued that this approach is incomplete and that apprehending the ethical issues requires a broader approach that considers social benefits as well as a more expanded consideration of animal welfare (DeGrazia and Beauchamp, 2021). Nevertheless, as with research with embryos, embryo models and human embryonic stem cells, proposed research with nonhuman animals is generally subject to oversight which considers the scientific objectives and minimizing harms to nonhuman animals where feasible.

Although minimizing moral incursions in research makes absolute sense, perhaps unexpectedly there is no consensus about how best to describe this obligation and its contours across different types of research. However, it is arguably important to provide reasons for making choices in morally contentious situations so that others can understand them and offer alternative options that may also be ethically sound. This seems especially true in life-science research where the implications of such choices broadly matter. As a practical matter, doing so is relevant throughout the life cycle of research from seeking approval from oversight bodies for proposed research and regulatory reviews as well as in grant proposals, scientific publications, and in public discussions.

Although minimizing moral incursions in research makes absolute sense, perhaps unexpectedly there is no consensus about how best to describe this obligation and its contours across different types of research.

## Misappropriation of the principle of subsidiarity

The “principle of subsidiarity” has curiously crept into ethics and policy deliberations about research with morally sensitive materials including embryos, stem cell-based embryo models, human pluripotent stem cells and assisted reproductive technologies to erroneously describe the obligation to minimize moral incursions (Pennings and Van Steirteghem, 2004; Bredenoord et al, 2017; Jans et al, 2018; Perreira-Daoud et al, 2020; Assen et al, 2021; Clark et al, 2021; Rivron et al, 2023; ESHRE et al, 2024). For example, it has been claimed that the “principle of subsidiarity states that one should not use an entity with a higher moral status if the research can be done using an entity with a lower moral status.” (ESHRE et al, 2024). However, this is a misnomer that does not cohere with standard definitions of the term subsidiarity, nor with well-established uses of this principle.

A simple dictionary definition of subsidiarity is “a principle in social organization holding that functions which are performed effectively by subordinate or local organizations belong more properly to them than to a dominant central organization.” The principle of subsidiarity—along with proportionality—has been central to governance in the EU since the outset: “The general aim of the principle of subsidiarity is to guarantee a degree of independence for a lower authority in relation to a higher body or for a local authority in relation to central government. It therefore involves the sharing of powers between several levels of authority, a principle which forms the institutional basis for federal states.” Similar interpretations can be found in social and political philosophy (Gosepath, 2005). Considered in this way, there are some potentially promising approaches to apply the principle of subsidiarity to bioethical issues, such as research with human subjects, personal health care and the implementation of public health programs (Kotalik, 2010).

In life-sciences research, the principle of subsidiarity properly understood actually captures some of the procedural and governance mechanisms used to address the associated ethical issues. This includes well-established mechanism such as oversight by research ethics committees/Institutional Review Boards, animal care and use committees, and stem-cell research oversight processes. Here, the principle of subsidiarity provides the ethical justification for making a range of choices within specified boundaries at a local level, which are sensitive to local laws, policies, and values; the authority has typically been designated by higher political authorities. These are critically important procedural mechanisms for making determinations in actual cases, operationalizing the principle of subsidiarity. Thus, there is a need for an alternative articulation of the obligation to minimizee moral incursions in research.

## Potential alternatives: proportionality and least infringement

There are at least two potential alternative constructs that might be appropriated to capture the obligation to take the least morally sensitive approach in research: the principle of proportionality and the condition of least infringement.

### The principle of proportionality

The principle of proportionality basically requires that for any given action, the good must outweigh the bad. The principle can have different and more granular expressions depending upon the context. For example, under the principle of proportionality, the EU requires that its “measures: must be suitable to achieve the desired end; must be necessary to achieve the desired end; and must not impose a burden on the individual that is excessive in relation to the objective sought to be achieved (proportionality in the narrow sense).” As described by Hermerén (2012), “The moral version of the principle of proportionality is about the relation between ends and means. The general idea underlying this principle seems to be that the relationship between ends and means should be ‘appropriate’ or ‘adequate’.”

The principle of proportionality has been used in some discussions regarding the ethical appropriateness of human pluripotent stem cell, embryo and stem cell-based embryo model research (Pennings and Van Steirteghem, 2004; Bredenoord et al, 2017; Jans et al, 2018; Perreira-Daoud et al, 2020; Assen et al, 2021; Clark et al, 2021). However, in this context the principle is typically used to assess the value of morally contentious research. For instance, “At the least, researchers will increasingly have to explain what kind of (and how much) tissue or cell source is necessary and proportionate, which means that the expected social value of the research should outweigh the moral harm of using an embryo or fetus for research.” (Bredenoord et al, 2017). Note that the term ‘social value’ derives from longstanding deliberations and international declarations primarily in the context of research with human subjects and is used to indicate that research must have scientific value in terms of enhancing knowledge or social value such as new medical treatments (Emanuel et al, 2000). In considering research in the life sciences involving morally sensitive materials under the principle of proportionality, the harm of a moral incursion must be weighed against the good of scientific or social value.

The principle of proportionality has been used in some discussions regarding the ethical appropriateness of human pluripotent stem cell, embryo and stem cell-based embryo model research.

In regard to research with human subjects, Hermerén (2012) finds that the principle of proportionality has four conditions: importance of the objective; relevance of means; most favourable option; and non-excessiveness. Under this conception, using the most favourable option might be considered as a plausible means of describing the need to minimize moral incursions in life-science research. However, it may be confounding to have the principle of proportionality carry the weight of both assessing social value of research compared to its harms as well as the somewhat separable obligation to minimize moral incursions in it.

### The condition of least infringement

The condition of “least infringement,” which has been used in one prominent approach to public health ethics (Childress et al, 2002), might be appropriated to describe the ethical obligation to minimize moral incursions in research. In public health, “when a policy infringes autonomy, public health agents should seek the least restrictive alternative; when it infringes privacy, they should seek the least intrusive alternative; and when it infringes confidentiality, they should disclose only the amount and kind of information needed, and only to those necessary, to realize the goal.” (Childress et al, 2002). In this approach to resolving conflicts among general moral considerations, least infringement is only one of five justificatory conditions, the others being effectiveness, proportionality, necessity, and public justification; here, proportionality is distinct from least infringement.

The condition of “least infringement,” […] might be appropriated to describe the ethical obligation to minimize moral incursions in research.

In the life sciences, the least infringement condition could be described as a requirement for relevant stakeholders, including scientists, sponsors, oversight bodies and regulators, to design and conduct research in such a way to minimize moral incursions to the extent possible without compromising scientific integrity. This requirement is separable from the need to justify proposed research according to relevant other considerations such as scientific value. That is, the least infringement condition is necessary, but not sufficient for determining the appropriateness of particular projects.

## Opportunities and challenges

Having a way to clearly describe the obligation to minimize moral incursions in research to accompany descriptions of its anticipated benefits is an important step especially given the *zeitgeist* cohering with this obligation. Nevertheless, any approach to minimizing moral incursions assumes broad agreement about the moral stakes at hand, such as the moral status of particular entities, which may not be the case. This needs to be considered and ideally made explicit as decisions are made about the justification for determining the appropriateness of particular research projects.

… any approach to minimizing moral incursions assumes broad agreement about the moral stakes at hand, such as the moral status of particular entities, which may not be the case.

Future work is needed to develop a robust and defensible approach to making appropriate determinations in particular cases. This would greatly help not only researchers but also regulators, ethics committees and review boards to properly balance moral incursions against the scientific and social value of research proposals. In the meantime, the least infringement condition seems to offer a reasonable articulation of some of the particularly salient concerns faced in research involving morally sensitive biological materials and nonhuman animals.

## Supplementary information

Peer Review File
