# Supplementary material for: Articulating the need to minimize moral incursions in research: The least infringement condition
Source: EMBO Rep. 2025 May 27;26(12):2973–6. doi: 10.1038/s44319-025-00490-w (PMC12187909; doi:10.1038/s44319-025-00490-w)
Supplement: Supplementary file 1 — Peer Review File [file 44319_2025_490_MOESM1_ESM.pdf]

EMBO reports

Articulating the need to minimize moral incursions in research. The Least Infringement condition.

Jeremy Sugarman

Corresponding author(s): Jeremy Sugarman (jsugarman@jhu.edu)

**Review Timeline:**	Submission Date:	20th May 25
	Accepted:	20th May 25

Editor: Holger Breithaupt

Transaction Report: This article was editorially reviewed.
